# Maternal hepatitis B surface antigen carrier status increased the incidence of gestational diabetes mellitus

**DOI:** 10.1186/s12879-019-3749-1

**Published:** 2019-02-13

**Authors:** Songxu Peng, Zhihua Wan, Xiaofang Lin, Xiu Li, Yukai Du

**Affiliations:** Department of Maternal and Child Health, School of Public Health, Tongji Medical College, Huazhong University of Science and Technology, 13th Hangkong Road, Wuhan, 430030 Hubei China

**Keywords:** Gestational diabetes mellitus, Hepatitis B virus infection, Hepatitis B surface antigen, Viral load

## Abstract

**Background:**

The relationship between chronic hepatitis B virus (HBV) infection with gestational diabetes mellitus (GDM) remains unclear. This study aimed to identify the association between maternal HBsAg-positive status and GDM.

**Methods:**

A retrospective cohort study was performed on the pregnant women who delivered from June 2012 to May 2016 at Wuhan Medical Care Center for Women and Children, Wuhan, China. We compared the incidence of GDM between HBsAg-positive pregnant women and HBsAg-negative controls. A multivariate regression model was used to measure the independent association between maternal HBsAg carrier and the risk of developing GDM.

**Results:**

In total, 964 HBsAg-positive pregnant women and 964 HBsAg-negative women were included into the study. We observed maternal HBsAg carrier (OR 1.47, 95% CI 1.06–2.03), age (OR 1.05, 95% CI 1.00–1.10) and family history of diabetes (OR 3.97, 95% CI 2.05–7.67) had an independent risk for GDM in multivariable logistical regression model. However, no significant association was found between HBeAg carrier status, other HBV markers or viral load in pregnancy and the incidence of GDM.

**Conclusions:**

Our results indicated that maternal HBsAg carriage is an independent risk factor for GDM, but viral activity indicated by HBeAg status and viral load is not the main reason for this phenomenon. Further studies are warranted to clarify the possible mechanisms behind such association of HBV infection and the additional risk of GDM.

**Electronic supplementary material:**

The online version of this article (10.1186/s12879-019-3749-1) contains supplementary material, which is available to authorized users.

## Background

Hepatitis B virus (HBV) infection is a major public health problem worldwide [1, 2]. With approximately 2 billion HBV-infected individuals around the world, more than 350 million persons have chronic HBV infection [3]. The global prevalence of HBV infection varies widely [4, 5]. However, the majority of hepatitis B infection patients live in Asia and Africa [6]. China is a highly endemic region, almost one third of chronic HBV carriers can be found there. The third national serological survey in 2006 shows that the hepatitis B surface antigen (HBsAg) positivity rate of Chinese aged 1–59 years is 7.18% [7]. Consequently, up to 10% of pregnant women are identified as chronic hepatitis B carrier in the screening for HBsAg status during the antenatal checkup, resulting in persistent HBV transmission [8, 9].

At present, a few studies have reported that HBV infection indicated by positive HBsAg status had a direct impact on pregnancy outcomes, such as threatened preterm labor, preterm birth, low birthweight, macrosomia, antepartum hemorrhage, pregnancy-induced hypertension [10–14]. However, the underlying mechanisms behind these associations have not yet been elucidated. As for the relationship between maternal HBsAg carrier and gestational diabetes mellitus (GDM), it remains controversial. Several studies have reported that chronic hepatitis B infection increased the risk of developing GDM [11, 15–17], and this result was confirmed by a latest study conducted by Lao et al. who performed a case-control study with 214 cases and 204 controls [18]. However, a meta-analysis by Kong et al. demonstrated that chronic hepatitis B surface antigenemia did not exert an additional risk for GDM in general population except Iranian [19]. Additionally, there are few studies that examined the effects of HBeAg status and maternal viral load in pregnancy on the development of GDM. In 2010, the new diagnostic criterion of GDM had been proposed due to associations between maternal hyperglycemia and adverse pregnancy outcomes [20]. Considering the high prevalence of chronic hepatitis B infection among pregnant women in China, it is extremely crucial to clarify whether or not HBsAg carrier status is associated with the risk of GDM.

Based on above, the objective of our study is not only to identify the correlation between HBsAg carrier and GDM, but also explore whether HBeAg status and maternal viral load in the third trimester are associated with an increased risk of GDM among HBsAg-positive pregnant women.

## Methods

### Study population

**Fig. 1:**
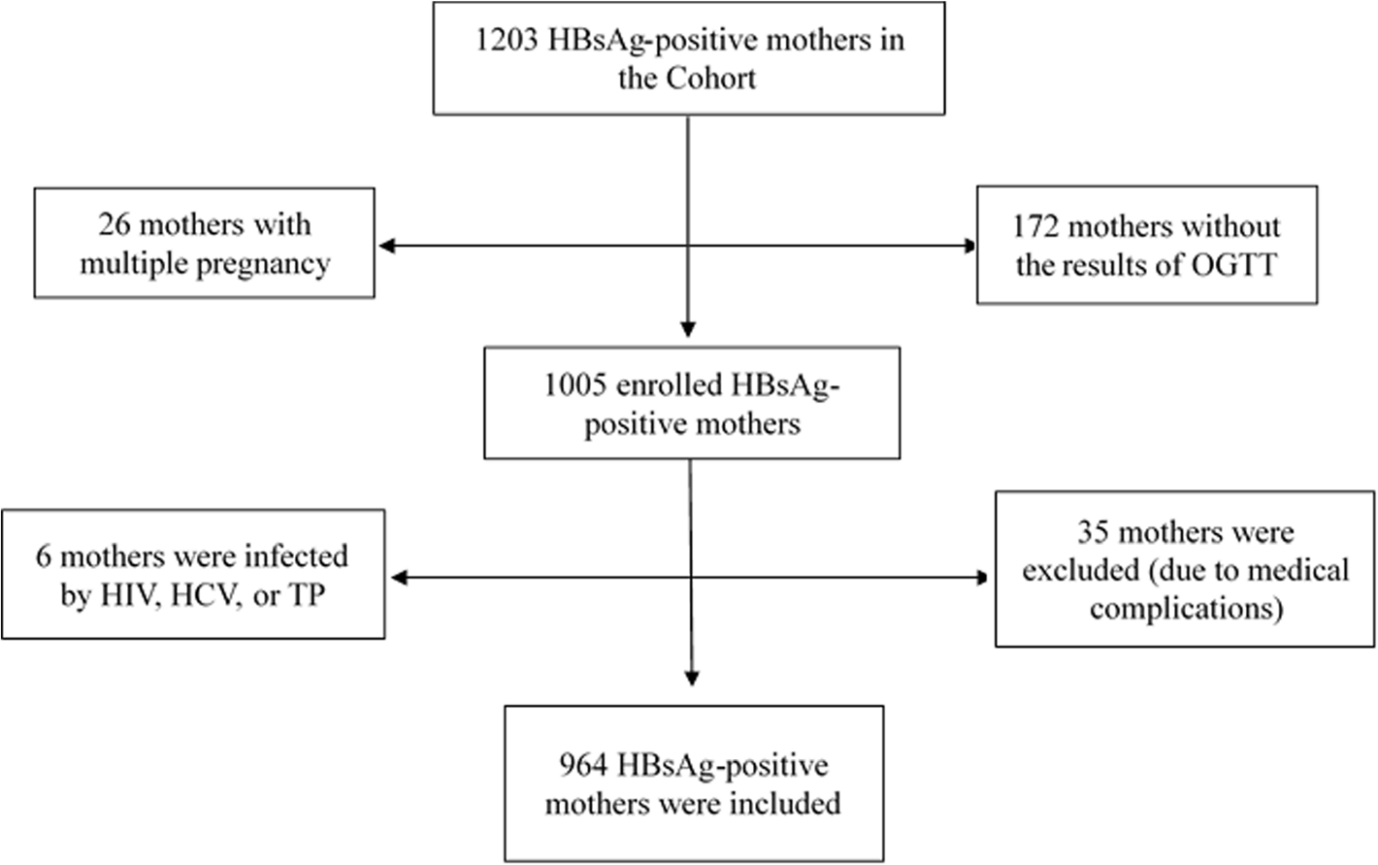
Flow chart of HBsAg-positive mothers selection. Abbreviations: HBsAg, hepatitisB surface antigen; OGTT, oral glucose tolerance test; HIV, human immunodeficiency virus; HCV, hepatitis C virus; TP, treponema pallidum

In the present study, the two groups of pregnant women including HBsAg-positive and HBsAg-negative mothers were recruited. HBsAg-positive pregnant women were from a pre-conception cohort in Wuhan which was designed to study the risk factors of HBV maternal-fetal transmission. HBsAg-positive pregnant women older than 20 who delivered from June 2012 to May 2016 were invited to participate in the cohort at Wuhan Medical Care Center for Women and Children, Wuhan, China. All HBsAg-positive women with singleton pregnancy and the complete results of oral glucose tolerance test (OGTT), who did not have current and previous medical complications (including HCV, HIV and Treponema pallidum infection), were assigned to HBsAg-positive group. A total of 964 HBsAg-positive women were eligible for the study (Fig. 1). Using the same criteria mentioned, the subjects of HBsAg-negative group were randomly chosen from electronic databases during the study period.

The present study was approved by the Institutional Review Board of Tongji Medical College, Huazhong University of Science and Technology. The written informed consent of all subjects had been obtained before participating the study.

### Data collection and samples collection

The clinical records of the two groups were retrieved for data extraction. The demographic information, including age, height, prenatal weight, parity, history of abortion, ABO blood type, family history of diabetes, and OGTT results were obtained from clinical records. We adopted the IADPSG criterion to diagnose GDM, that is, the pregnant women were diagnosed with GDM during 24th -28th weeks of gestation when their OGTT results exceeded the following glucose values: fasting plasma glucose level > 5.1 mmol/L and/or 1-h plasma glucose level > 10 mmol/L and/or 2-h plasma glucose level > 8.5 mmol/L.

For all HBsAg-positive women, a venous blood sample had been collected in the third trimester once informed consent had been obtained. Blood samples were used to examine anti-HBsAg, HBeAg, anti-HBeAg, anti-HBcAg status and maternal viral load. Serum HBV markers were tested by enzyme-linked immune sorbent assay (Kehua Biotechnology, Shanghai, China). The hepatitis B viral load was quantified by fluorescence quantitative polymerase chain reaction (FQ-PCR) (Da’an Gene Co. Ltd., Sun Yat-Sen University, Guangdong, China). All procedures were performed strictly following the manufacturer’s instructions.

### Statistical analysis

In univariate analyses, continuous variables were analyzed by Student’s t-tests and categorical data were compared by chi-square tests. Stratified analyses were used to identify confounders and effect modifiers, and the Breslow-Day test was used to assess the homogeneity of the odds ratios (ORs) for HBsAg carriage across each stratum of risk factors. Odds ratios and 95% confidence intervals (CIs) were estimated using multivariable logistical regression to measure the association between maternal HBsAg status and GDM. The variables were chosen according to their statistical and clinical relevance. The stepwise (Wald) method was used in the multivariate logistic regression analysis. Statistical significance was assessed at the 5% level (two-tail test). All analyses were performed using SPSS software version 18.0 (SPSS, Chicago, IL, USA).

## Results

A total of 964 HBsAg-positive women and 964 HBsAg-negative women, according with the inclusion criteria, were enrolled during June 2012 to May 2016. In this study, the highest proportion of missing data is 4.8% for prenatal BMI, and the missing proportion of age, prenatal weight, height, parity, ABO type, history of abortion and family history of diabetes were 0.6, 4.2, 2.8, 0.5, 1.5, 2.4 and 3.7%, respectively. Because the missing rates are small, we did not perform an additional process for the missing data. The maternal characteristics of the study population and the incidence of GDM are presented in Table 1. Compared with HBsAg-negative mothers, HBsAg-positive women had a significantly higher age, proportion of multiparas and history of abortion. But there was no significant difference in prenatal weight, height, or calculated body mass index (BMI) between mothers in HBsAg-positive group and those in HBsAg-negative group. Neither the distribution of ABO blood types nor family history of diabetes showed statistical difference in the two groups.

**Table 1:** Maternal characteristics and incidence of GDM with respect to maternal HBsAg status

Characteristics	HBsAg status		*P*-value
	Positive (964)	Negative (964)	
Age (years)	29.17 ± 4.02	28.26 ± 3.25	**< 0.001**
Age ≥35 years (%)^a^	11.5	5.0	**< 0.001**
Prenatal weight (kg)	70.56 ± 9.31	70.29 ± 10.14	0.587
Height (cm)	161.40 ± 4.60	161.24 + 4.69	0.488
Prenatal BMI (kg/m^2^)	27.08 ± 3.35	27.01 ± 3.59	0.710
BMI ≥ 28 kg/m^2^ (%) ^a^	31.1	29.9	0.570
Multiparas (%) ^a^	20.0	13.8	**0.001**
ABO type (%) ^a^			0.624
A	32.7	32.5	
B	26.0	27.8	
O	32.3	30.0	
AB	9.0	9.7	
History of abortion (%) ^a^	41.3	34.4	**0.003**
Family history of diabetes (%) ^a^	2.7	3.2	0.553
GDM (%) ^a^	16.5	10.5	< **0.001**

*Abbreviation*: *BMI* Body mass index, *GDM* Gestational diabetes mellitus

^a^Chi-square test for categorical variables

The present study found that HBsAg carriers were more likely to suffer from GDM as compared to HBsAg-negative women (16.5% vs 10.5%, *P <* 0.001, Table 1). To determine the interactions between each of known factors affecting the development of GDM and maternal HBsAg status, stratified analyses by HBsAg status were performed according to each of these factors, i.e. age ≥ or < 35 years, nullipara or multipara, the presence or absence of family history of diabetes, with or without history of abortion. As shown in Table 2, positive HBsAg status was associated with an increased risk of GDM in women aged < 35 years, having no family history of diabetes, with an odds ratio of 1.60(95% CI 1.19–2.15) and 1.73(95% CI 1.27–2.23), respectively. Regardless of the parity and the status of history of abortion, positive HBsAg status exerted an additional risk for GDM and the differences reached statistical significance. However, when we used the Breslow-Day test to assess the homogeneity of the ORs for HBsAg carriage across each stratum of risk factors, no significant difference was seen in these stratification factors.

**Table 2:** Incidence of GDM with respect to HBsAg status in pregnant women, stratified by risk factors

Factors	GDM (%)		*P*-value	OR	95% CI	*P-*Value ^a^
	HBsAg+	HBsAg-				
Age < 35 years	15.5	10.3	**0.002**	1.60	1.19–2.15	0.758
Age ≥ 35 years	24.3	14.6	0.198	1.88	0.71–4.94	
Nullipara	15.4	10.8	**0.009**	1.51	1.11–2.05	0.125
Multipara	21.4	8.8	**0.005**	2.80	1.34–5.84	
Family history of diabetes	46.2	34.6	0.397	1.62	0.53–4.95	0.911
No family history of diabetes	15.7	9.7	**< 0.001**	1.73	1.27–2.23	
History of abortion	18.8	12.8	**0.035**	1.59	1.03–2.44	0.811
No history of abortion	14.8	9.3	**0.005**	1.70	1.17–2.47	

*Abbreviation*: *GDM* Gestational diabetes mellitus, *OR* Odds ratio, *CI* Confidence interval

^a^*P*-values for interaction effect between each risk factor and HBsAg status on GDM

In univariate analyses, age and HBsAg carriage were associated with the increased incidence of GDM, with an OR value of 1.08(95% CI 1.04–1.11) and 1.67(95% CI 1.27–2.23), respectively. Family history of diabetes (OR 4.60, 95% CI 2.60–8.14) and history of abortion (OR 1.38, 95% CI 1.05–1.81) increased the incidence of GDM. But there was no significant difference in terms of parity and prenatal BMI between the mothers with GDM and those without. To determine whether HBsAg carriage was an independent risk factor for GDM, multivariable logistic regression analysis was performed, adjusting for the confounding effects of other factors that were also found to be significantly different between the two groups (age, parity, history of abortion) or considered as classical risk factors of GDM in previous study (prenatal BMI, family history of diabetes). Age, prenatal BMI, parity as continuous variables and history of abortion, family history of diabetes as categorical variables were included in the multivariable logistic regression analysis. After adjustment for these associated covariates, a significant association of maternal HBsAg carriage with the increased risk of GDM was observed (OR 1.47, 95% CI 1.06–2.03) (Table 3). Simultaneously, significant associations between age as well as family history of diabetes and the increased risk of GDM were detected. However, there was no significant association between the risk for GDM and other factors including prenatal BMI, parity, history of abortion.

**Table 3:** Univariate and multivariate logistic regression analyses of factors related to GDM

Variable	Univariate		Multivariate	
	*OR (95% CI)*	*P*-value	*OR (95% CI)*	*P*-value
Age	1.08 (1.04–1.11)	**< 0.001**	1.05 (1.00–1.10)	**0.044**
Prenatal BMI	1.02 (0.97–1.06)	0.448	1.00 (0.96–1.05)	0.948
Parity	1.30 (0.93–1.82)	0.122	1.08 (0.71–1.65)	0.717
HBsAg	1.67 (1.27–2.23)	**< 0.001**	1.47 (1.06–2.03)	**0.021**
Family history of diabetes	4.60 (2.60–8.14)	**< 0.001**	3.97 (2.05–7.67)	**< 0.001**
History of abortion	1.38 (1.05–1.81)	**0.019**	1.27 (0.91–1.76)	0.159

*Abbreviation*: *OR* Odds ratio, *CI* Confidence interval

In the multivariable logistic regression analysis, the association between HBsAg carriage and the risk of GDM remained significant after adjustment for other covariates. Further analysis was performed to explore whether other HBV markers and maternal viral load were associated with the risk of GDM among HBsAg-positive pregnant women. As a result, no significant relationship was observed between HBeAg status and incidence of GDM among HBsAg carriers (data shown in Additional file 1: Tables S1). Similarly, the various antibodies to HBsAg, HBeAg, HBcAg, did not show any significant association with the incidence of GDM. Among HBsAg-positive pregnant women, the distribution of viral load in third trimester were not significantly different in women with and without GDM.

## Discussion

Our study found an independent effect of maternal HBsAg carriage on GDM, and noted pregnant women with HBV infection had an increased risk of GDM, confirming the findings of a previous study [15]. When maternal age was taken into account, maternal HBV infection increased the risk of GDM in the mothers younger than 35 years of age, similar to that exerted on pregnant women without family history of diabetes. When parity or history of abortion was examined, HBsAg carriage increased the risk of GDM in nulliparous or multiparous women, and similar results could be obtained in the women with or without history of abortion. Despite of no significant difference in Breslow-Day test, it can be speculated that there were interactions between maternal HBsAg carriage and other maternal factors, which resulted in various risk for GDM in pregnant women. The results of this study showed that the risk of GDM heightened with the increase of age. Additionally, our study also indicated that the incidence of GDM increased in the pregnancy women with higher age or family history of diabetes, implying that increasing age and genetic factor were the important contributors to the development of GDM [21].

In the few published studies regarding the effects of HBV infection on pregnancy outcomes, some reported a positive association between HBV infection and the risk of GDM [11, 15–18]. Among them, several retrospective studies done by Lao et al. suggested HBsAg carrier was significantly associated with gestational diabetes mellitus [15, 16]. Our present study demonstrated HBsAg-positive pregnant women were more likely to suffer from GDM (OR 1.43, 95%CI 1.01–2.02) compared with HBsAg-negative mothers. However, this result was contradicted by many others, which supported the hypothesis that women with HBV infection did not have extra risk for GDM [12–14, 22]. This inconsistent result could be related to ethnic difference. Because the prevalence of HBV infection and genetic background differ in various ethnic groups [23, 24]. This may affect the actual association of HBsAg carriage and the risk of GDM. Additionally, the present study adopted the IADPSG criterion, and then more pregnant women were diagnosed with GDM.

Although some researchers noticed the correlation between HBV infection and GDM, and attempted to clarify the intrinsic links, the potential mechanism for this association is unclear. In addition, as shown in our study, different degree of viral activity indicated by HBeAg status and viral load in pregnancy could not explain the higher risk of GDM in HBV-infected women. Several previous reviews and reports established that the pathogenesis of GDM was related to insulin resistance associated with the chronic inflammatory state [25, 26]. In addition to the effects of the pregnancy itself, the factors involved in the inflammatory state were elevated levels of pro-inflammatory cytokines such as IL-2, IL-6, IL-10, and tumor necrosis factor-alpha (TNF-a), which can be accounted for by chronic HBV infection [27]. Second, some patients with HBV infection developed into liver fibrosis or cirrhosis, which has been identified as a cause of insulin resistance and glucose intolerance [28]. Third possible mechanism is the increased iron status caused by chronic HBV infection facilitates the development of GDM [16]. The main reason is that excess iron can affect insulin synthesis and secretion, facilitate oxidation of lipids and liver mediated insulin resistance [29, 30].

The strengths of the present study include the large number of subjects and the use of multivariable logistic regression analysis for adjusting potential confounding variables. Additionally, this study comprehensively explore the associations between HBV markers, viral load in pregnancy and GDM despite no statistically significant difference presented. However, the limitations of our study is also unavoidable. The first and most obvious limitation is that our study is a retrospective study that proved a positive correlation between HBsAg carrierage and GDM. But its capability of etiological inferences is limited. Therefore a large-scale prospective study on this causal relationship is needed. Second, we have only the data of prenatal maternal weight, and the pre-gravid maternal weight was missing in this study. Thereby we could not calculate the BMI before conception despite of its impact on the development of GDM. This might affect the authenticity of our results to a certain extent.

## Conclusion

HBV infection can lightly increase the incidence of GDM and viral activity may not be the main reason generating this phenomenon. In view of unknown pathophysiologic mechanism of GDM and high prevalence of HBV infection in certain regions, further research in this area should obviously be needed to explain our present epidemiological observation.

## Additional file

Additional file 1: **Table S1.** The association between HBV markers, maternal viral load and GDM in HBsAg-positive pregnant women. (DOCX 19 kb)

## Abbreviations

BMI: Body mass index
CI: Confidence interval
GDM: Gestational diabetes mellitus
HBeAg: Hepatitis B envelope antigen
HBsAg: Hepatitis B surface antigen
HBV: Hepatitis B virus
HCV: Hepatitis C virus
HIV: Human immunodeficiency virus
IADPSG: International Association of Diabetes and Pregnancy Study Groups
OGTT: Oral glucose tolerance test
OR: Odds ratio
TP: Treponema pallidum
