# Supplementary material for: Maternal hepatitis B surface antigen carrier status increased the incidence of gestational diabetes mellitus
Source: BMC Infect Dis. 2019 Feb 13;19:147. doi: 10.1186/s12879-019-3749-1 (PMC6373004; doi:10.1186/s12879-019-3749-1)
Supplement: Supplementary file 1 — Table S1. The association between HBV markers, maternal viral load and GDM in HBsAg-positive pregnant women. (DOCX 19 kb) [file 12879_2019_3749_MOESM1_ESM.docx]

**Table S1** The association between HBV markers, maternal viral load and GDM in HBsAg-positive pregnant women

| Items | GDM (n=159) | non-GDM (n=805) | *P*-value | OR (95%CI) |
| --- | --- | --- | --- | --- |
| HBeAg+ (%) | 16.8 (26/155) | 18.5 (147/794) | 0.608 | 1.13 (0.71-1.78) |
| Anti-HBsAg+ (%) | 12.9 (20/155) | 10.2 (81/794) | 0.318 | 0.77 (0.46-1.06) |
| Anti-HBeAg+ (%) | 72.2 (112/154) | 74.8 (594/794) | 0.477 | 1.15 (0.78-1.69) |
| Anti-HBcAg+ (%) | 94.8 (147/155) | 95.6 (758/794) | 0.737 | 1.14 (0.52-2.51) |
| Viral load in the second trimester | |  |  |  |
| ≤10^3^ copies/ml | 69.8 (37/53) | 67.7 (193/285) | | 1.00 (Reference) |
| 10^3^-10^6^ copies/ml | 22.6 (12/53) | 19.3 (55/285) | 0.303 | 1.77 (0.60-5.27) |
| ≥ 10^6^ copies/ml | 7.5 (4/53) | 13.0 (37/285) | 0.254 | 2.02 (0.61-6.74) |
| Viral load in the third trimester | |  |  |  |
| ≤10^3^ copies/ml | 61.9 (60/97) | 64.1 (352/549) |  | 1.00 (Reference) |
| 10^3^-10^6^ copies/ml | 23.7 (23/97) | 21.1 (116/549) | 0.966 | 0.99 (0.53-1.85) |
| ≥ 106 copies/ml | 14.4 (14/97) | 14.8 (81/549) | 0.710 | 1.15 (0.56-2.36) |

GDM, Gestational diabetes mellitus; OR, odds ratio; CI, confidence interval.
